# Supplementary material for: Synthesis, Crystal Structure, and Characterization of Energetic Salts Based on 3,5-Diamino-4H-Pyrazol-4-One Oxime
Source: Molecules. 2023 Jan 3;28(1):457. doi: 10.3390/molecules28010457 (PMC9824549; doi:10.3390/molecules28010457)
Supplement: Supplementary file 1 [file molecules-28-00457-s001.zip › molecules-2117560-supplementary.pdf]

Supporting information

Synthesis, Crystal Structure, and Characterization of Energetic Salts Based on 3,5-Diamino-4*H*-Pyrazol-4-One Oxime

Wen-Shuai Dong [1], Lu Zhang [1], Wen-Li Cao [1,2], Zu-Jia Lu [1], Qamar-un-Nisa Tariq [1], Chao Zhang [1], Xiao-Wei Wu [1], Zong-You Li [1] and Jian-Guo Zhang [1,*]

[1] State Key Laboratory of Explosion Science and Technology, Beijing Institute of Technology, Beijing 100081, China

[2] China Ordnance Society, Beijing 100089, China

* Correspondence: zjgbit@bit.edu.cn; Tel.: +86-10-68918091

Section S1. Crystal structure data

Table S1. Bond Lengths for DAPO

Atom	Atom	Length/Å	Atom	Atom	Length/Å
N1	N2	1.431(3)	N4	C2	1.323(4)
N1	C3	1.329(4)	N5	C3	1.330(4)
N2	C1	1.310(4)	C1	C2	1.481(4)
N3	C1	1.361(4)	C2	C3	1.463(4)
N4	O1	1.315(3)			

Table S2. Bond Angles for DAPO

Atom	Atom	Atom	Angle/°	Atom	Atom	Atom	Angle/°
C3	N1	N2	113.9(2)	N4	C2	C1	133.2(3)
C1	N2	N1	105.0(2)	N4	C2	C3	123.4(3)
O1	N4	C2	116.5(2)	C3	C2	C1	103.4(2)
N2	C1	N3	123.1(3)	N1	C3	N5	124.8(3)
N2	C1	C2	111.8(3)	N1	C3	C2	105.9(3)
N3	C1	C2	125.2(3)	N5	C3	C2	129.2(3)

Table S3. Torsion Angles for DAPO

A	B	C	D	Angle/°	A	B	C	D	Angle/°
N1	N2	C1	N3	178.7(3)	N4	C2	C3	N1	178.6(3)
N1	N2	C1	C2	-0.4(3)	N4	C2	C3	N5	-1.3(5)
N2	N1	C3	N5	179.2(3)	O1	N4	C2	C1	-1.9(5)
N2	N1	C3	C2	-0.7(3)	O1	N4	C2	C3	-179.4(3)
N2	C1	C2	N4	-177.9(3)	C1	C2	C3	N1	0.4(3)
N2	C1	C2	C3	0.0(4)	C1	C2	C3	N5	-179.5(3)
N3	C1	C2	N4	3.0(6)	C3	N1	N2	C1	0.7(3)
N3	C1	C2	C3	-179.1(3)					

Table S4. Bond Lengths for DAPOC

Atom	Atom	Length/Å	Atom	Atom	Length/Å
N2	C3	1.330(4)	N3	C2	1.285(3)
O1	N3	1.359(3)	C3	N5	1.305(3)
N4	N5	1.425(3)	C3	C2	1.480(4)
N4	C1	1.316(3)	C2	C1	1.468(4)
N1	C1	1.313(4)			

Table S5. Bond Angles for DAPOC

Atom	Atom	Atom	Angle/°	Atom	Atom	Atom	Angle/°
C1	N4	N5	113.2(2)	N3	C2	C3	134.7(3)
C2	N3	O1	112.9(2)	N3	C2	C1	121.6(2)
N2	C3	C2	124.8(2)	C1	C2	C3	103.7(2)
N5	C3	N2	124.8(3)	N4	C1	C2	106.1(2)
N5	C3	C2	110.3(2)	N1	C1	N4	127.3(2)
C3	N5	N4	106.6(2)	N1	C1	C2	126.6(2

Table S6. Torsion Angles for DAPOC

A	B	C	D	Angle/°	A	B	C	D	Angle/°
N2	C3	N5	N4	-178.2(3)	C3	C2	C1	N1	179.4(3)
N2	C3	C2	N3	-0.1(5)	N5	N4	C1	N1	-179.0(2)
N2	C3	C2	C1	178.1(3)	N5	N4	C1	C2	-0.3(3)
O1	N3	C2	C3	-1.6(4)	N5	C3	C2	N3	-178.9(3)
O1	N3	C2	C1	-179.4(2)	N5	C3	C2	C1	-0.8(3)
N3	C2	C1	N4	179.0(2)	C2	C3	N5	N4	0.6(3)
N3	C2	C1	N1	-2.2(4)	C1	N4	N5	C3	-0.2(3)
C3	C2	C1	N4	0.6(3)					

Table S7. Bond Lengths for DAPOP

Atom	Atom	Length/Å	Atom	Atom	Length/Å
Cl1	O3	1.436(5)	N4	C2	1.296(6)
Cl1	O4	1.419(5)	N5	C3	1.313(8)
Cl1	O5	1.431(5)	N6	N7	1.427(7)
Cl1	O6	1.450(5)	N6	C6	1.323(6)
Cl2	O7	1.417(5)	N7	C4	1.324(7)
Cl2	O8	1.417(6)	N8	C4	1.302(7)
Cl2	O9	1.432(6)	N9	O2	1.339(6)
Cl2	O10	1.423(5)	N9	C5	1.287(6)
N1	N2	1.407(7)	N10	C6	1.325(7)
N1	C3	1.323(7)	C1	C2	1.493(6)
N2	C1	1.324(6)	C2	C3	1.478(7)
N3	C1	1.342(7)	C4	C5	1.473(7)
N4	O1	1.336(5)	C5	C6	1.486(7)

Table S8. Bond Angles for DAPOP

Atom	Atom	Atom	Angle/°	Atom	Atom	Atom	Angle/°
O3	Cl1	O6	108.7(3)	N2	C1	N3	124.9(4)
O4	Cl1	O3	109.2(3)	N2	C1	C2	110.0(4)
O4	Cl1	O5	111.2(4)	N3	C1	C2	125.1(4)
O4	Cl1	O6	110.2(3)	N4	C2	C1	133.0(5)
O5	Cl1	O3	107.8(4)	N4	C2	C3	123.6(5)
O5	Cl1	O6	109.7(3)	C3	C2	C1	103.4(4)
O7	Cl2	O8	105.1(4)	N1	C3	C2	105.6(5)
O7	Cl2	O9	111.3(4)	N5	C3	N1	127.8(5)
O7	Cl2	O10	111.8(4)	N5	C3	C2	126.5(5)
O8	Cl2	O9	107.9(5)	N7	C4	C5	105.7(5)
O8	Cl2	O10	107.6(4)	N8	C4	N7	128.8(5)
O10	Cl2	O9	112.7(5)	N8	C4	C5	125.5(5)
C3	N1	N2	114.5(4)	N9	C5	C4	122.7(5)
C1	N2	N1	106.4(4)	N9	C5	C6	133.4(5)

C2	N4	O1	112.4(4)	C4	C5	C6	104.0(4)
C6	N6	N7	106.0(4)	N6	C6	N10	124.2(5)
C4	N7	N6	114.0(4)	N6	C6	C5	110.3(5)
C5	N9	O2	114.0(4)	N10	C6	C5	125.5(4)

Table S9. Torsion Angles for DAPOP

A	B	C	D	Angle/°	A	B	C	D	Angle/°
N1	N2	C1	N3	180.0(5)	N7	C4	C5	C6	0.1(5)
N1	N2	C1	C2	0.8(6)	N8	C4	C5	N9	-0.1(9)
N2	N1	C3	N5	178.6(6)	N8	C4	C5	C6	178.7(5)
N2	N1	C3	C2	1.2(6)	N9	C5	C6	N6	179.2(5)
N2	C1	C2	N4	179.6(5)	N9	C5	C6	N10	0.4(9)
N2	C1	C2	C3	-0.1(6)	O1	N4	C2	C1	0.2(8)
N3	C1	C2	N4	0.4(9)	O1	N4	C2	C3	179.9(5)
N3	C1	C2	C3	-179.3(5)	O2	N9	C5	C4	179.5(4)
N4	C2	C3	N1	179.6(5)	O2	N9	C5	C6	1.1(8)
N4	C2	C3	N5	2.1(9)	C1	C2	C3	N1	-0.6(6)
N6	N7	C4	N8	-179.3(6)	C1	C2	C3	N5	-178.1(6)
N6	N7	C4	C5	-0.7(6)	C3	N1	N2	C1	-1.3(6)
N7	N6	C6	N10	177.8(5)	C4	C5	C6	N6	0.6(5)
N7	N6	C6	C5	-1.0(6)	C4	C5	C6	N10	-178.2(5)
N7	C4	C5	N9	-178.7(5)	C6	N6	N7	C4	1.1(6)

Table S10. Bond Lengths for DAPOT

Atom	Atom	Length/Å	Atom	Atom	Length/Å
O1	C4	1.225(8)	N5	C3	1.276(8)
O2	N6	1.185(8)	N6	C5	1.478(8)
O3	N6	1.202(8)	N7	C7	1.449(9)
O4	N7	1.234(8)	N8	C9	1.452(9)
O5	N7	1.222(8)	C1	C2	1.471(10)
O6	N8	1.214(8)	C2	C3	1.474(9)

O7	N8	1.195(7)	C4	C5	1.414(9)
O11	N4	1.335(7)	C4	C9	1.479(9)
N1	N2	1.382(8)	C5	C6	1.361(9)
N1	C3	1.320(8)	C6	C7	1.367(10)
N2	C1	1.292(9)	C7	C8	1.354(10)
N3	C1	1.319(9)	C8	C9	1.370(10)
N4	C2	1.268(9)			

Table S11. Bond Angles for DAPOT

Atom	Atom	Atom	Angle/°	Atom	Atom	Atom	Angle/°
C3	N1	N2	113.9(5)	N5	C3	N1	126.6(6)
C1	N2	N1	108.0(6)	C2	C3	N1	104.7(6)
C2	N4	O11	112.8(6)	C2	C3	N5	128.6(7)
O3	N6	O2	122.3(7)	C5	C4	O1	125.9(6)
C5	N6	O2	119.6(6)	C9	C4	O1	122.1(6)
C5	N6	O3	118.1(7)	C9	C4	C5	112.0(6)
O5	N7	O4	122.7(7)	C4	C5	N6	119.7(6)
C7	N7	O4	117.9(7)	C6	C5	N6	115.3(6)
C7	N7	O5	119.3(8)	C6	C5	C4	125.0(6)
O7	N8	O6	122.6(7)	C7	C6	C5	118.8(7)
C9	N8	O6	115.6(7)	C6	C7	N7	119.1(7)
C9	N8	O7	121.8(6)	C8	C7	N7	118.6(7)
N3	C1	N2	128.7(7)	C8	C7	C6	122.2(7)
C2	C1	N2	109.7(6)	C9	C8	C7	119.6(7)
C2	C1	N3	121.6(7)	C4	C9	N8	119.8(6)
C1	C2	N4	124.7(7)	C8	C9	N8	117.8(7)
C3	C2	N4	131.6(7)	C8	C9	C4	122.3(7)
C3	C2	C1	103.7(6)				

Table S12. Torsion Angles for DAPOT

A	B	C	D	Angle/°	A	B	C	D	Angle/°
O1	C4	C5	N6	0	N1	N2	C1	C2	0
O1	C4	C5	C6	180	N1	C3	C2	N4	180
O1	C4	C9	N8	0	N1	C3	C2	C1	0
O1	C4	C9	C8	180	N2	C1	C2	N4	180
O2	N6	C5	C4	0	N2	C1	C2	C3	0
O2	N6	C5	C6	180	N3	C1	C2	N4	0
O3	N6	C5	C4	180	N3	C1	C2	C3	180
O3	N6	C5	C6	0	N4	C2	C3	N5	0
O4	N7	C7	C6	0	N5	C3	C2	C1	180
O4	N7	C7	C8	180	N6	C5	C4	C9	180
O5	N7	C7	C6	180	N6	C5	C6	C7	180
O5	N7	C7	C8	0	N7	C7	C6	C5	180
O6	N8	C9	C4	180	N7	C7	C8	C9	180
O6	N8	C9	C8	0	N8	C9	C4	C5	180
O7	N8	C9	C4	0	N8	C9	C8	C7	180
O7	N8	C9	C8	180	C4	C5	C6	C7	0
O11	N4	C2	C1	180	C4	C9	C8	C7	0
O11	N4	C2	C3	0	C5	C6	C7	C8	0
N1	N2	C1	N3	180	C6	C7	C8	C9	0

Section S2. Calculation of formation enthalpy

Firstly, the enthalpy of formation of neutral molecule is required. The molecular formula of DAPO is $C_3H_5N_5O$. According to the definition of enthalpy of formation, the enthalpy of formation of DAPO is equal to its enthalpy minus the enthalpy of the most stable element. The calculation formula is as follows:

$$\Delta_f H_m^{\theta}(\mathrm{M}) = H(\mathrm{M}) - 3H\big(\mathrm{C(g)}\big) - \frac{5}{2}H(\mathrm{H_2}) - \frac{5}{2}H(\mathrm{N_2}) - \frac{1}{2}H(\mathrm{O_2}) + 3\big(\mathrm{C(s)}\big)$$

The above enthalpy values are calculated quantitatively. All structures were optimized under B3PW91/6-31G (d, p), and then calculated under PWPB95D3/def2-QZVPP with high precision single

point energy. After zero-point energy correction, their enthalpy values were obtained respectively. Because the steady state of carbon in the standard state is the solid phase, the enthalpy of the gas phase is quantified, so the standard molar enthalpy of sublimation of the carbon needs to be added. Then, according to the enthalpy of formation of the neutral molecule, the enthalpy of formation of its cation M^+ can be calculated, and the calculation formula is as follows:

$$\Delta_f H_m^\theta(\mathrm{M}^+) = \Delta_f H_m^\theta(\mathrm{M}) - \Delta_f H_m^\theta(\mathrm{H}^+) - H(\mathrm{M}) + H(\mathrm{H}^+) + H(\mathrm{M}^+)$$

Then, according to the empirical formula, the lattice energy of the ionic salt ΔH_L was calculated:

$$U_{POT}/\mathrm{KJ\ mol^{-1}}=\gamma(\rho_m/M_m)^{1/3}+\sigma$$

$$\Delta H_L=U_{POT}+[p(\eta_M/2-2)+q(\eta_X/2-2)]/RT$$

Finally, according to the Born-Haber cycle, the enthalpy of formation of ionic salts can be calculated as follows:

$$\Delta_f H_m^\theta(salt) = \Delta_f H_m^\theta(\mathrm{cation}) + \Delta_f H_m^\theta(anion) - \Delta H_L$$

The anions involved in the calculation of DAPOP and DAPOT are perchlorate and picrate, and their formation enthalpies are calculated using the G4 thermodynamic combination method in a process similar to the calculation of cation formation enthalpies. The enthalpy of formation of protons is derived from the National Institute of Standards and Technology (NIST).
